# Correction: Efficient NIR energy conversion of plasmonic silver nanostructures fabricated with the laser-assisted synthetic approach for endodontic applications

**DOI:** 10.1039/d0ra90117b

**Published:** 2020-11-11

**Authors:** Tetiana Bulavinets, Magdalena Kulpa-Greszta, Anna Tomaszewska, Malgorzata Kus-Liśkiewicz, Gabriela Bielatowicz, Iryna Yaremchuk, Adriana Barylyak, Yaroslav Bobitski, Robert Pązik

**Affiliations:** Department of Photonics, Lviv Polytechnic National University S. Bandera Str. 12 79013 Lviv Ukraine tetiana.o.bulavinets@lpnu.ua; Faculty of Chemistry, Rzeszow University of Technology Aleja Powstańców Warszawy 12 35-959 Rzeszow Poland; Department of Biotechnology, Institute of Biology and Biotechnology, College of Natural Sciences, University of Rzeszow Pigonia 1 35-310 Rzeszow Poland; Department of Therapeutic Dentistry, Danylo Galytsky Lviv National Medical University Pekarska Str., 69 79010 Lviv Ukraine; Department of Physics, Centre of Microelectronic and Nanotechnology, College of Natural Sciences, University of Rzeszow Pigonia 1 35-310 Rzeszow Poland

## Abstract

Correction for ‘Efficient NIR energy conversion of plasmonic silver nanostructures fabricated with the laser-assisted synthetic approach for endodontic applications’ by Tetiana Bulavinets *et al.*, *RSC Adv.*, 2020, **10**, 38861–38872, DOI: 10.1039/D0RA06614A.

The authors regret that the name of one of the authors (Anna Tomaszewska) was shown incorrectly in the original article. The corrected author list is as shown above.

The Royal Society of Chemistry apologises for these errors and any consequent inconvenience to authors and readers.

## Supplementary Material

